# Risk signals and clinical characteristics of serious adverse events of neutropenia associated with pembrolizumab: a FAERS database study

**DOI:** 10.1186/s12885-026-15630-1

**Published:** 2026-01-24

**Authors:** Jianhui Niu, Shanshan Xu

**Affiliations:** Department of Pharmacy, Beijing Tongren Hospital, Capital Medical University, Beijing, China

**Keywords:** Pembrolizumab, Neutropenia, FAERS, Pharmacovigilance, Immune checkpoint inhibitor (ICI), Time-to-onset (TTO)

## Abstract

**Background:**

This study aimed to characterize the risk signals and clinical features of pembrolizumab-associated neutropenia reported as serious adverse events (SAEs) in the US FDA Adverse Event Reporting System (FAERS), and the association between concomitant chemotherapy and time-to-onset.

**Methods:**

In this retrospective pharmacovigilance study, SAE reports from FAERS (2021Q1–2024Q2) with pembrolizumab designated as the primary suspect drug were analyzed. Among 14,747 pembrolizumab-associated SAE reports, 856 with the Preferred Term “neutropenia” were identified and characterized. Clinical characteristics were summarized, and subgroup analyses explored associations between concomitant chemotherapy and TTO, as well as between sex and the reporting odds ratio (ROR). A supplementary analysis of febrile neutropenia (FN) cases was conducted to assess clinical severity.

**Results:**

Among 14,747 SAEs, 856 were neutropenia cases, for which a strong signal of disproportional reporting was detected (ROR = 5.23, 95% CI: 4.88–5.60). Median patient age was 61.0 years, and the reported mortality rate was 19.4%. A higher ROR was observed in females (7.06) than males (4.42), a difference likely influenced by the underlying distribution of cancer types and the 9.8% missing data for sex, rather than indicating an intrinsic sex-related risk. The overall median TTO was 19.0 days, with the concomitant chemotherapy group (N = 268) showing a shorter median TTO (14.5 days) than the non-chemotherapy group (N = 55, 39.0 days; p = 0.0006). Among 311 FN cases, 86.5% (269/311) occurred in the concomitant chemotherapy group.

**Conclusion:**

Pembrolizumab-associated neutropenia represents a notable safety signal in FAERS. The observed early-onset pattern, particularly in the context of concomitant chemotherapy, provides real-world evidence characterizing the clinical features of this adverse event.

## Introduction

Pembrolizumab, an immune checkpoint inhibitor (ICI), has demonstrated significant clinical benefit across various malignancies [1–3]. However, its unique spectrum of immune-related adverse events (irAEs) presents new safety challenges [4]. Among these irAEs, serious hematologic toxicities, particularly neutropenia—which can increase the risk of potentially fatal infections—represent a critical clinical challenge, potentially compromising treatment continuity and patient safety [5]. While the precise mechanisms underlying ICI-related hematologic toxicities remain incompletely understood, immune-mediated processes such as T-cell–driven injury to hematopoietic progenitor cells or autoantibody production have been hypothesized [6]. Characterizing the real-world risk profile of serious events related to this adverse reaction is therefore essential for optimizing clinical risk management [7].

The US FDA Adverse Event Reporting System (FAERS) provides a valuable resource for investigating rare but serious adverse reactions [8, 9]. While previous studies have characterized ICI-related hematologic toxicities, there is limited real-world data describing the clinical characteristics of pembrolizumab-associated neutropenia, particularly regarding its time-to-onset (TTO) and the potential influence of concomitant chemotherapy. This study therefore aimed to systematically characterize the clinical features and risk signals of neutropenia reported as a serious adverse event with pembrolizumab in the FAERS database, with a focus on TTO patterns and the impact of concomitant chemotherapy, thereby providing real-world evidence to inform clinical safety management.

## Materials and methods

### Data source and study cohort

This study utilized data from the publicly available US FDA Adverse Event Reporting System (FAERS) and extracted all quarterly data files from Q1 2021 to Q2 2024 [10].

To establish our analytical cohorts, we included all reports where pembrolizumab was designated as the “Primary Suspect” drug and the event was classified as a Serious Adverse Event (SAE) according to FDA criteria. An SAE is defined as any event that results in death, is life-threatening, requires or prolongs hospitalization, causes a persistent or significant disability/incapacity, results in a congenital anomaly/birth defect, or is another important medical event that may require intervention to prevent permanent impairment [11]. To ensure data quality, duplicate reports were removed, retaining only the latest version of each unique safety report ID. This process resulted in a final overall analysis cohort for disproportionality analysis, from which a core cohort of reports with neutropenia classified as a serious adverse event was identified for clinical characterization.

### Coding and processing of adverse events and drug information

All adverse events in this study were coded according to the Medical Dictionary for Regulatory Activities (MedDRA, Version 28.0) [12]. All signal detection and clinical characterization analyses were conducted at the Preferred Term (PT) level to ensure precision and consistency. Specifically, to ensure the homogeneity of the reported adverse event phenotype for the primary TTO and signal analyses, the core study cohort was strictly defined using the single MedDRA PT ‘Neutropenia’ (*N* = 856). However, given the critical clinical implication of febrile neutropenia as a medical emergency, we conducted a separate, supplementary descriptive analysis on the PT ‘Febrile neutropenia’. These cases were analyzed specifically to assess the association between this severe outcome and concomitant chemotherapy, but were excluded from the primary neutropenia cohort to avoid duplicate counting and heterogeneity.

To improve data consistency, we standardized the text data for “medicinalproduct” and “drugindication”. This process involved converting all text to uppercase, removing irrelevant punctuation, and mapping common synonyms or variant spellings (e.g., various expressions of “Non-small cell lung cancer”) to a standardized term.

### Signal detection and subgroup analysis

To assess for disproportionality in reporting between pembrolizumab and specific adverse events, we employed four standard disproportionality analysis algorithms [13]: the Reporting Odds Ratio (ROR), the Proportional Reporting Ratio (PRR), the Information Component (IC), and the Empirical Bayes Geometric Mean (EBGM). All calculations were based on a 2 × 2 contingency table, where ‘a’ represents the number of reports in which pembrolizumab was identified as the primary suspect (PS) drug for the target event (e.g., neutropenia); ‘b’ is the number of reports of all other events for pembrolizumab as the PS drug; ‘c’ is the number of reports for the target event involving all other drugs (excluding pembrolizumab); ‘d’ is the number of reports of all other events for all other drugs (excluding pembrolizumab).

Internationally recognized criteria were applied to identify positive signals [14, 15]. A signal was considered present if the respective criteria were met: (1) for ROR, the lower limit of the 95% confidence interval (CI) was greater than 1; (2) for PRR, the value was ≥ 2 with at least 3 cases and a chi-square value of ≥ 4; (3) for IC, the lower limit of the 95% CI was greater than 0; and (4) for EBGM, the lower limit of the 90% CI (EBGM05) was greater than 2.

Furthermore, sex-stratified disproportionality analyses were performed within the core neutropenia cohort (*N* = 856), with RORs and their 95% CIs calculated for each subgroup.

### Clinical characterization

We conducted a systematic analysis of the clinical characteristics of the core neutropenia cohort (*N* = 856), extracting and processing the following variables:

#### Demographic and baseline characteristics

We extracted patient age and sex. Age was reported as median and interquartile range (IQR).

#### Temporal characteristics

Time-to-onset (TTO) was a key endpoint of this study. To calculate TTO as accurately as possible while maximizing the sample size, we established the following hierarchical strategy:

1. Primary method: For cases where both “drugstartdate” and “reactionstartdate” were recorded, TTO was calculated as the difference in days between these two dates.
2. Secondary method: For cases missing this date information, we estimated the TTO by converting the reported “drugtreatmentduration” and its corresponding unit. Non-positive values and extreme outliers, which were considered biologically implausible, were excluded from the analysis.

#### Clinical outcomes

To assess the severity of the adverse event, we extracted and tabulated the number and percentage of cases reported with the following serious outcomes as defined by FAERS: death, life-threatening, or hospitalization (including initial or prolonged). It should be noted that these outcome categories are not mutually exclusive, and a single case report may be associated with more than one outcome.

#### Medical context

1. Primary Tumor Indications: We precisely identified the specific indications for pembrolizumab by algorithmically matching the drug name, its “drugcharacterization” field (set as “Primary Suspect”), and the indication within each report. The primary tumor types were then extracted, categorized and ranked by frequency.

2) Concomitant Medications: To describe the most frequently co-administered medications, we extracted all drugs reported as “concomitant” or “interacting” with pembrolizumab. After standardizing the drug names, the top 10 most frequently reported concomitant medications were tabulated.

Furthermore, to explore the association between concomitant chemotherapy and TTO, we conducted a subgroup analysis on the 323 cases with available TTO data. Based on the concomitant medication information, we categorized cases into two groups: (a) Concomitant Chemotherapy group: cases where at least one pre-defined cytotoxic chemotherapy drug was listed as a concomitant medication. This list included carboplatin, cisplatin, paclitaxel, docetaxel, nab-paclitaxel, pemetrexed, gemcitabine, cyclophosphamide, etoposide, fluorouracil, oxaliplatin, irinotecan, vinorelbine, and doxorubicin. (b) No Concomitant Chemotherapy group: cases where none of the aforementioned chemotherapy drugs were listed.

### Statistical analysis

All data processing and statistical analyses in this study were performed using Python (version 3.9; Python Software Foundation) and its core scientific computing libraries, primarily pandas for data manipulation, and SciPy for statistical testing.

Descriptive statistics were used to summarize the baseline characteristics and clinical outcomes of the study cohort: continuous variables were described using medians and interquartile ranges (IQRs), while categorical variables were described using frequencies (n) and percentages (%). The non-parametric Mann-Whitney U test was used to compare median TTO between the concomitant chemotherapy subgroups. Missing data were addressed using an available-case analysis strategy, with the sample size (N) explicitly reported for each analysis.

## Results

### Study cohort and risk signals

**Fig. 1:**
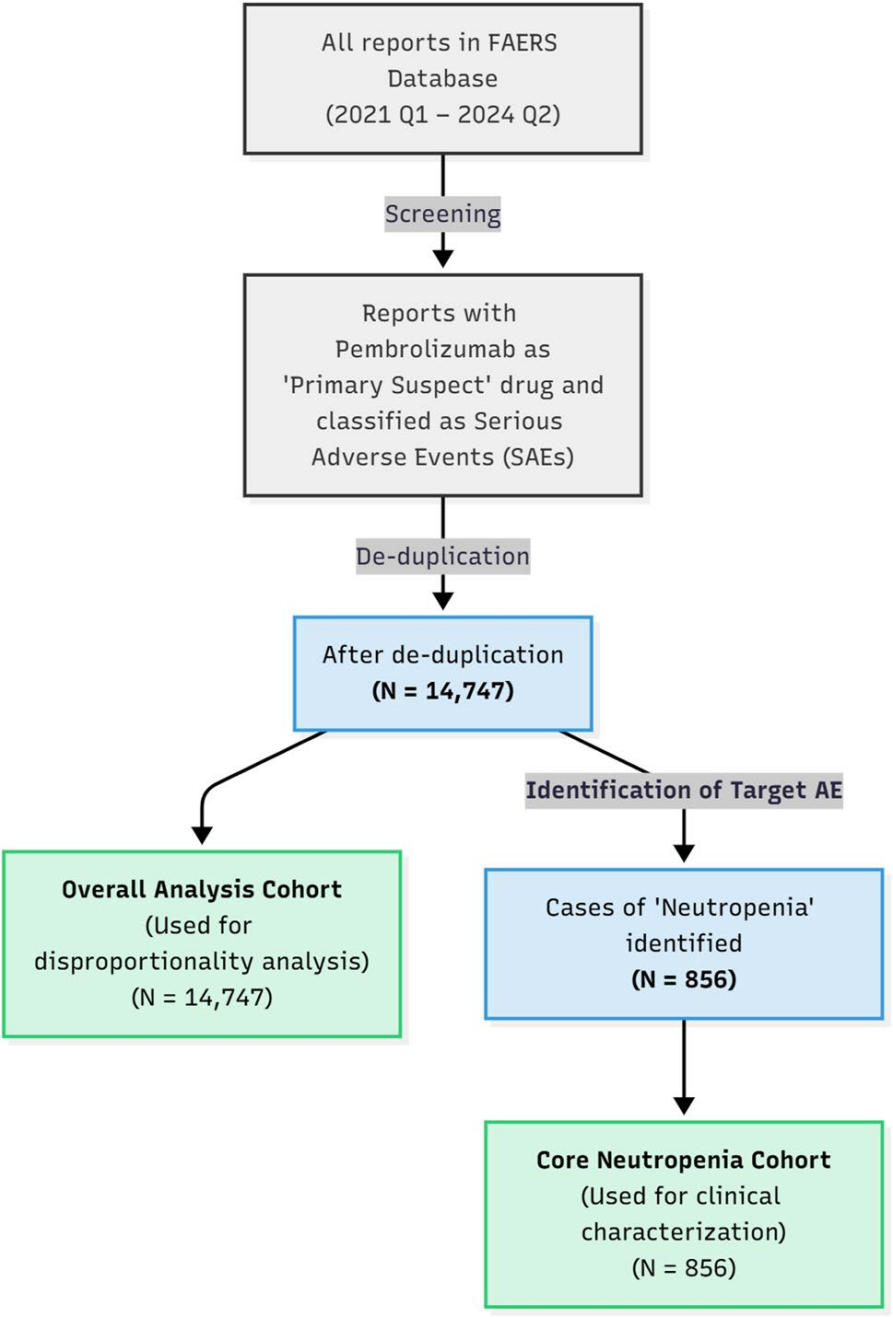
Flowchart of study cohort selection. The diagram illustrates the process of screening the FAERS database to identify the overall analysis cohort (*N* = 14,747) used for disproportionality analysis, and the core neutropenia cohort (*N* = 856) used for all subsequent clinical characterization

The cohort selection process for this study is shown in Fig. 1. From a total of 14,747 serious adverse event (SAE) reports associated with pembrolizumab, 856 reports of neutropenia were identified.

Disproportionality analysis of the overall cohort was performed to screen for safety signals. The results revealed strong signals of disproportional reporting for multiple hematologic toxicities (Table 1). Among these, neutropenia emerged as the most frequently reported hematologic SAE (*n* = 856), representing a strong safety signal across all four disproportionality analysis algorithms.

**Table 1:** Signal detection results for hematologic serious adverse events associated with pembrolizumab

Adverse Event (Preferred Term)	Reports (*n*)	ROR (95% CI)	PRR (χ²)	IC (95% CI)	EBGM (95% CI)
Neutropenia	856	5.23 (4.88–5.60)	4.99 (2720.70)	2.31 (2.25–2.37)	4.99 (4.67–5.33)
Pancytopenia	355	10.28 (9.25–11.43)	10.06 (2822.09)	3.31 (3.19–3.42)	10.04 (9.04–11.12)
Thrombocytopenia	447	5.39 (4.90–5.92)	5.26 (1527.76)	2.38 (2.30–2.47)	5.26 (4.80–5.76)

### Clinical characteristics and serious clinical outcomes of neutropenia reports

The demographic characteristics and clinical outcomes of the 856 neutropenia cases included in the final analysis are summarized in Table 2.

**Table 2:** Clinical characteristics, outcomes, and sex-stratified subgroup analysis of neutropenia cases associated with pembrolizumab (*N* = 856)

Characteristic	Statistic
Demographics	
Age (years), median (IQR) (*N* = 721)^a^	61.0 (50.0–72.0)
Age ≥ 65 years, n (%)	302 (41.9%)
Sex, n (%) (*N* = 772)^b^	
Female	398 (51.6%)
Male	374 (48.4%)
Clinical Outcomes, n (%) (*N* = 856)	
Hospitalization (caused/prolonged)	399 (46.6%)
Life-threatening	104 (12.1%)
Death	166 (19.4%)
Subgroup analysis of reporting odds ratio (ROR) by sex	
Female (*n* = 398)	7.06 (95% CI: 6.40–7.79)
Male (*n* = 374)	4.42 (95% CI: 4.01–4.88)

The sex-stratified ROR analysis is exploratory and likely confounded by the distribution of cancer types (e.g., TNBC) and treatment regimens

CI Confidence Interval

^a^Age information was missing in 135 cases (15.8%)

^b^Sex information was missing in 84 cases (9.8%)

**Fig. 2:**
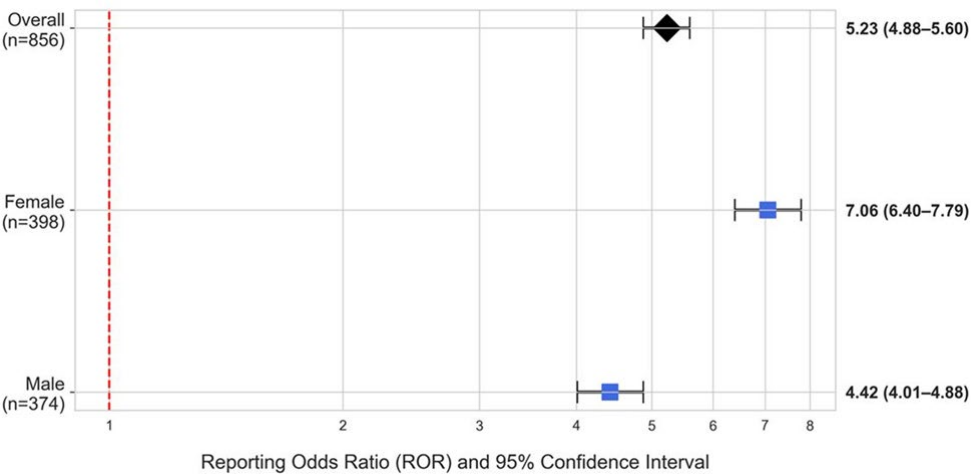
Sex-stratified subgroup analysis of the reporting odds ratio (ROR) for neutropenia associated with pembrolizumab. The forest plot shows the RORs and their 95% confidence intervals (CIs) for neutropenia associated with pembrolizumab, stratified by sex. Squares represent the point estimates for each subgroup, the diamond represents the overall point estimate, and the horizontal lines represent the 95% CIs. The red dashed line indicates the line of no effect (ROR = 1)

Among the 721 cases with available age information, the median patient age was 61.0 years (IQR: 50.0–72.0). Of the 772 cases with available sex information, 51.6% were female. An exploratory sex-stratified disproportionality analysis showed a numerically higher ROR for neutropenia in the female subgroup (ROR = 7.06, 95% CI: 6.40–7.79) compared to the male subgroup (ROR = 4.42, 95% CI: 4.01–4.88), with no overlap between their respective 95% CIs (Fig. 2). However, this finding should be interpreted with caution, as it does not account for potential confounding factors, such as differences in underlying cancer types, or the uncertainty introduced by the 9.8% of cases with missing sex information.

The clinical outcomes associated with neutropenia included hospitalization in 46.6% of cases and an overall mortality rate of 19.4%.

### TTO analysis

Among all 856 cases, a total of 323 (37.7%) reports contained sufficient information to calculate the TTO.

**Fig. 3:**
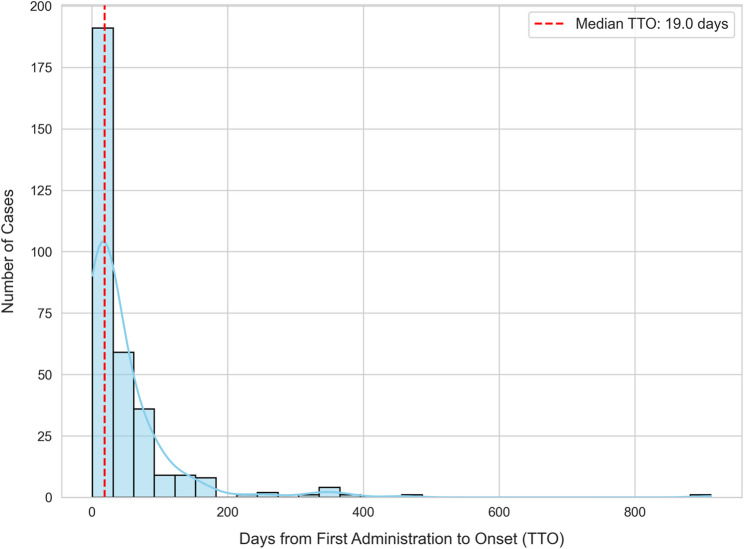
Distribution of time-to-onset (TTO) for neutropenia associated with pembrolizumab (*N* = 323). The histogram shows the frequency distribution of TTO, with the red dashed line indicating the median TTO (19.0 days)

**Fig. 4:**
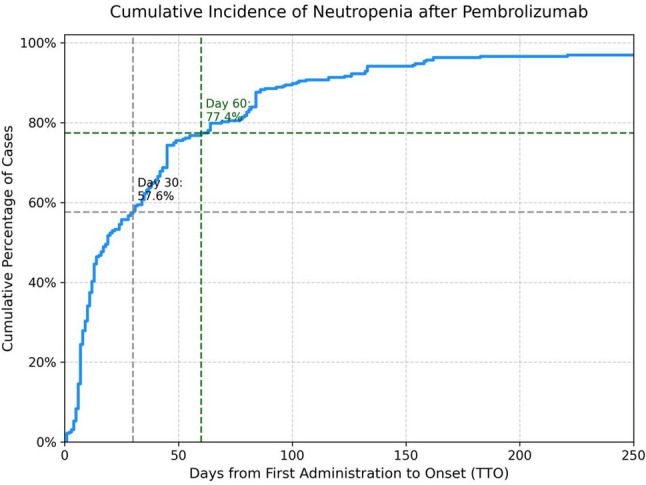
Cumulative proportion curve of time-to-onset (TTO) for neutropenia associated with pembrolizumab (*N* = 323). The cumulative curve illustrates the percentage of events accumulating over time. The gray dotted and green dashed lines indicate the cumulative proportions at day 30 (57.6%) and day 60 (77.4%) after treatment initiation, respectively

The median TTO for neutropenia was 19.0 days (IQR: 8.0–48.5). The temporal characteristics of these reported events are presented in Figs. 3 and 4. The histogram in Fig. 3 shows a right-skewed frequency distribution, with a high number of cases reported in the early phase of treatment. The cumulative incidence curve in Fig. 4 illustrates this early-onset pattern quantitatively: 57.6% of events occurred within the first 30 days of treatment initiation, and more than three-quarters (77.4%) occurred within the first 60 days.

### Association between concomitant chemotherapy and TTO

**Fig. 5:**
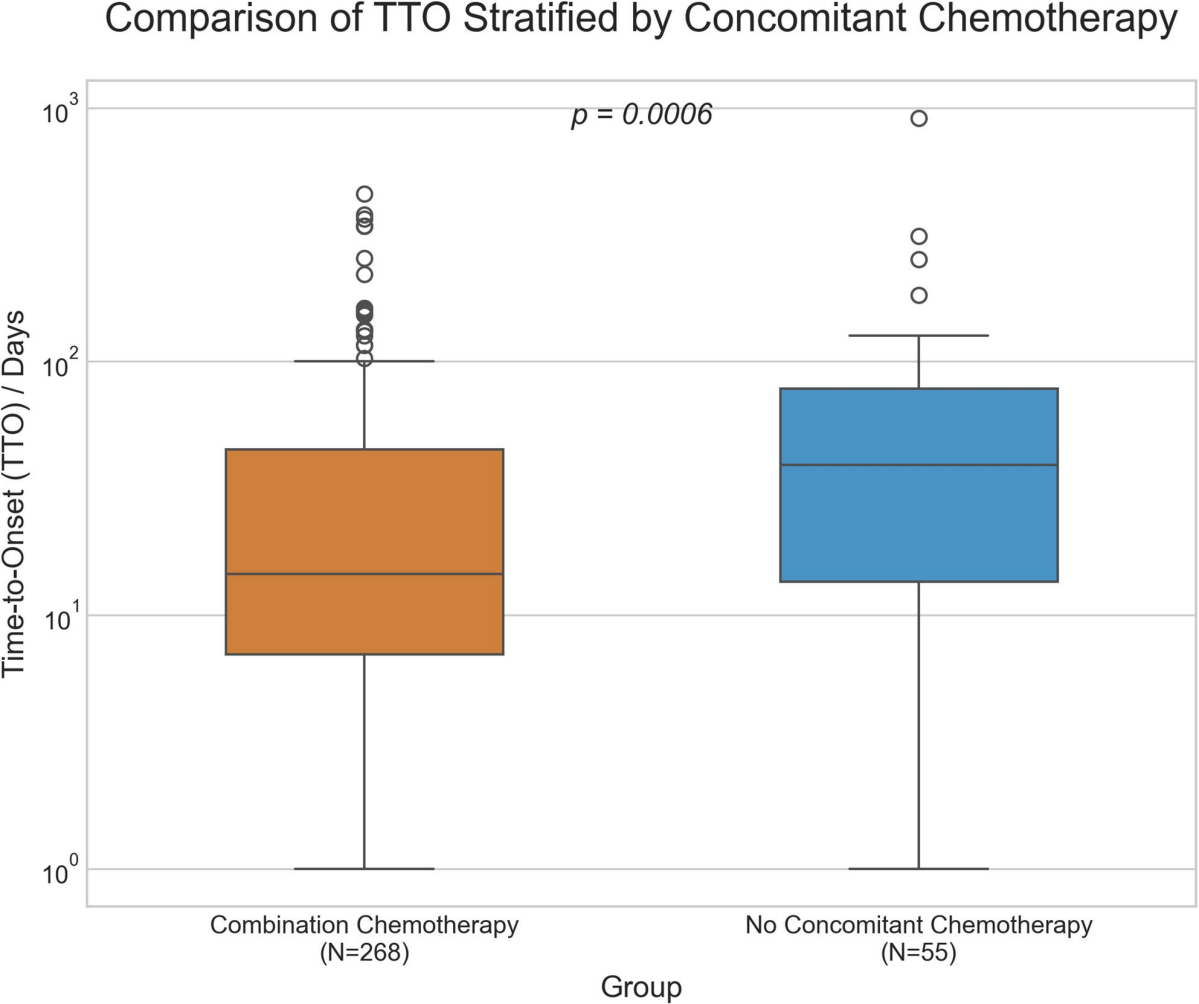
Comparison of time-to-onset (TTO) for neutropenia, stratified by concomitant chemotherapy. The box plot displays the median, interquartile range (IQR), and distribution of TTO for the Concomitant Chemotherapy group (*N* = 268) and the No Concomitant Chemotherapy group (*N* = 55). The Y-axis is presented on a logarithmic scale to better visualize the data distribution. The difference between the two groups is statistically significant (Mann-Whitney U test, *p* = 0.0006)

Of the 323 cases with evaluable TTO, a vast majority (268, 83.0%) were reported as having received concomitant chemotherapy. Subgroup analysis revealed a statistically significant difference in TTO between the groups with and without concomitant chemotherapy (Fig. 5).

The median TTO for the Concomitant Chemotherapy group (268, 83.0%) was 14.5 days (IQR: 7.0–45.0), which was significantly shorter than the 39.0 days (IQR: 13.5–78.0) observed in the No Concomitant Chemotherapy group (*N* = 55, 17.0%). This difference was confirmed by the Mann-Whitney U test (U = 5328.0, *p* = 0.0006).

### Common primary tumors and concomitant medications

Analysis of patients’ medical background (Table 3) showed that the most common tumor treated with pembrolizumab was non-small cell lung cancer, reported in 16.1% of cases (*n* = 138), followed by triple-negative breast cancer (11.0%, *n* = 94).

**Table 3:** Top 5 specific indications for pembrolizumab and top 10 most common concomitant medications (*N* = 856)

Rank	Indication^a^	Cases, *n* (%)^b^	Concomitant Drug	Cases, *n* (%)
1	Non-small cell lung cancer (NSCLC)	138 (16.1%)	Carboplatin	542 (63.3%)
2	Triple-negative breast cancer (TNBC)	94 (11.0%)	Pemetrexed	298 (34.8%)
3	Lung adenocarcinoma	53 (6.2%)	Paclitaxel	224 (26.2%)
4	Malignant lung neoplasm	49 (5.7%)	Dexamethasone	101 (11.8%)
5	Breast cancer	39 (4.6%)	Cisplatin	99 (11.6%)
6			Cyclophosphamide	81 (9.5%)
7			Ondansetron	73 (8.5%)
8			Acetaminophen	72 (8.4%)
9			Folic acid	61 (7.1%)
10			Lenvatinib	50 (5.8%)

^a^The list of indications excludes “UNKNOWN INDICATION” (*n* = 61) and non-oncology indications (e.g., “PROPHYLAXIS”, *n* = 11)

^b^Percentages were calculated based on the total number of cases (*N* = 856)

Regarding concomitant medications, several cytotoxic chemotherapy drugs were frequently reported, indicating that combination chemotherapy is a prevalent treatment approach. Among these chemotherapy agents, carboplatin was the most commonly reported (63.3%, *n* = 542), followed by pemetrexed (34.8%, *n* = 298).

### Supplementary analysis of febrile neutropenia

To further evaluate the clinical relevance of the signal, we performed a supplementary search for the Preferred Term ‘Febrile neutropenia’ within the overall cohort (*N* = 14,747) and identified 311 cases. Consistent with the pattern observed in the neutropenia cohort, the majority of these serious febrile events (*n* = 269, 86.5%) occurred in patients receiving concomitant chemotherapy, reflecting the distribution of serious hematologic events in the context of combination therapy.

## Discussion

A central finding of this study is that a significant majority of reports of neutropenia classified as serious adverse events associated with pembrolizumab occurred in the context of concomitant chemotherapy. This is highlighted by the fact that 83.0% of cases in our TTO cohort received concurrent chemotherapy. This context is critical for interpreting all subsequent findings. The safety signal and clinical characteristics described herein likely reflect the combined toxicity profile of chemo-immunotherapy combination regimens as a whole, rather than an effect attributable to pembrolizumab alone. While pembrolizumab was designated the “Primary Suspect,” the profound myelosuppressive effect of cytotoxic chemotherapy is likely a major contributing factor. This study, therefore, aims not to assign causality but to characterize the real-world safety profile of this prevalent therapeutic combination.

In our study cohort, the median age of patients with reports of neutropenia classified as serious adverse events was 61.0 years. This age distribution is likely influenced by the unique cancer type composition of our cohort. Specifically, lung cancer and breast cancer were the most predominant tumor types. Lung cancer, being a malignancy that typically occurs in older populations (median age at diagnosis ~ 71 years) [16], raised the overall age baseline of the patients. However, the proportion of triple-negative breast cancer (TNBC) was notably high in the cohort (11.0%). Given its higher prevalence in younger women [17], TNBC may explain why our cohort’s median age (61 years) was lower than that of the general breast cancer population (63 years) [16]. Nevertheless, over 40% of the cases still occurred in patients aged 65 or older, which again highlights the dominant role of lung cancer as the primary indication.

Our exploratory analysis identified a higher reporting odds ratio (ROR) for neutropenia in females compared to males. However, this observation must be interpreted with extreme caution as it is likely driven by significant confounding by indication, and further limited by uncertainty from missing data (9.8%). Notably, triple-negative breast cancer (TNBC), a disease exclusive to women, was the second most frequent malignancy in our cohort. The standard-of-care for TNBC often involves pembrolizumab combined with aggressive, myelosuppressive chemotherapy. Therefore, the elevated ROR in the female subgroup very likely reflects the concentrated use of these specific high-risk combination regimens, rather than an intrinsic biological sex difference. While some studies, such as the one by Unger et al., have suggested a higher risk of severe adverse events in females receiving immunotherapy [18], our finding, given these strong limitations, cannot be used to support that conclusion in this context.

The serious clinical outcomes observed in this cohort warrant attention: a mortality rate of 19.4% and a hospitalization rate of 46.6% underscore the seriousness of these events. Because FAERS lacks detailed clinical information, it is difficult to determine whether these outcomes were directly caused by neutropenia or instead reflect underlying disease progression or comorbid conditions. Nevertheless, the observed proportions suggest that neutropenia reported in association with pembrolizumab may represent a serious adverse event with important clinical consequences [19–21]. These results indicate that recognizing and characterizing the clinical presentation patterns of this event may hold value for future risk-management strategies.

The overall median time-to-onset (TTO) observed in this study was 19.0 days (interquartile range: 8.0–48.5), notably shorter than the previously reported median TTO for ICI-related neutropenia (approximately 45 days, or 6.4 weeks) [22]. Subgroup analysis revealed a significant difference in TTO between patients receiving concomitant chemotherapy and those who did not: the median TTO was 14.5 days for the concomitant chemotherapy group (*n* = 268, 83.0%) versus 39.0 days for the non-chemotherapy group (*n* = 55, 17.0%) (*p* = 0.0006). Given that combination chemotherapy was the predominant treatment modality in the TTO cohort (83.0%), this subgroup finding largely accounts for the overall early-onset pattern. Although the specific drivers of this early-onset pattern cannot be determined, these observations underscore the variation in neutropenia onset across different treatment contexts.

In this study, a supplementary analysis of febrile neutropenia (FN) cases in the FAERS database revealed that 86.5% (269/311) of FN events occurred in patients receiving concomitant chemotherapy. Although we were unable to directly link the time to onset of neutropenia with the occurrence of FN at the individual patient level, the concordant distribution of both events within the high-risk context of combination chemotherapy may reflect the clinical relevance of the TTO findings. Notably, in the pivotal KEYNOTE-189 trial, the incidence of severe (Grade 3–5) neutropenia was numerically higher in the pembrolizumab-plus-chemotherapy group compared with the placebo-plus-chemotherapy group (16.3% vs. 11.9%) [23], further highlighting neutropenia as a clinically relevant safety concern in the context of combination therapy. Although these datasets are not directly comparable, our TTO observations emphasize early-onset characteristics of neutropenia in real-world settings, providing supportive evidence for understanding the clinical profile of this adverse event.

This study has several important limitations that must be considered when interpreting the results. First, and most critically, this study is susceptible to confounding by indication and other unmeasured variables inherent to the FAERS database. We were unable to access or adjust for crucial patient-level clinical data, such as prior lines of chemotherapy, previous radiation therapy, performance status, cancer stage, or the presence of bone marrow metastases, all of which are independent risk factors for neutropenia. Second, as a spontaneous reporting system, FAERS is subject to well-known biases, including under-reporting, reporting bias (Weber effect), and variable data quality. For example, critical information for TTO calculation was missing in over 60% of our initial cohort. Third, the observational design precludes any determination of causality; all findings, particularly the disproportionality signals, should be interpreted as associations requiring further validation. The designation of pembrolizumab as the ‘Primary Suspect’ may also be influenced by reporter bias, especially in the highly complex context of combination chemotherapy. Fourth, the database has limitations in data granularity. It lacks standardized adverse event grading (e.g., CTCAE), preventing a more refined severity analysis. Importantly, in the FAERS database, the designation of a serious adverse event reflects regulatory reporting criteria rather than standardized clinical severity grading, and thus should not be interpreted as equivalent to grade-defined severe neutropenia. We were also unable to systematically assess the incidence of febrile neutropenia, a more specific indicator of clinical severity, due to inconsistent reporting terminology. Similarly, a direct causal link between the neutropenia event and the reported severe outcomes (e.g., death) cannot be established, as these could be attributable to disease progression or other comorbidities. Finally, it should be noted that our disproportionality analysis compares reports of the target adverse event for pembrolizumab (as the primary suspect) against all other events for all other drugs in FAERS, including reports where pembrolizumab was a non-primary suspect. Therefore, the detected signals indicate relative reporting disproportionality within the FAERS database, rather than comparative risk versus other anticancer therapies.

## Conclusion

This study systematically analyzed the characteristics of neutropenia reported as serious adverse events associated with pembrolizumab in the FAERS database. The results revealed observed disproportionality signals and illustrated their association with severe clinical outcomes. Notably, the time-to-onset analysis showed a shorter median onset time for neutropenia in the context of concomitant chemotherapy. These observations provide real-world evidence that may contribute to a better understanding of the clinical features of this adverse event.

## Data Availability

The datasets analyzed during the current study are publicly available in the FDA Adverse Event Reporting System (FAERS) repository, available at: [https://www.fda.gov/drugs/questions-and-answers-fdas-adverse-event-reporting-system-faers/fda-adverse-event-reporting-system-faers-public-dashboard].
